# The Thai version of the COVID-19 Yorkshire Rehabilitation Scale: a valid instrument for the psychometric assessment of the community members in Bangkok, Thailand

**DOI:** 10.1186/s12889-023-15566-2

**Published:** 2023-04-11

**Authors:** Suphamas Partiprajak, Suphanna Krongthaeo, Noppawan Piaseu, Jatuporn Wongsathikun, Anon Kongsuwan

**Affiliations:** 1 Ramathibodi School of Nursing, Faculty of Medicine Ramathibodi Hospital, Mahidol University, 270 Rama VI Road, Phayathai, Rachathewi, Bangkok, 10400 Thailand; 2 Cardiovascular Pulmonary Physical Therapy Unit, Chaopraya Hospital, Borommaratchachonnani Road, Arun Amarin, Bangkoknoi, Bangkok, 10700 Thailand; 3 Center for Health Promotion and Well-being, Faculty of Medicine Ramathibodi Hospital, Mahidol University, 270 Rama VI Road, Phayathai, Rachathewi, Bangkok, 10400 Thailand

**Keywords:** COVID-19 Yorkshire Rehabilitation Scale, C19-YRS, Long-term COVID, Psychometric properties, Thailand, Translation

## Abstract

**Background:**

Coronavirus disease 2019 (COVID-19) can develop into a long-term COVID in some cases, which can have a major impact on various health systems requiring appropriate treatment involving multi-disciplinary healthcare. The COVID-19 Yorkshire Rehabilitation Scale (C19-YRS) is a standardized tool widely used for screening the symptoms and severity of long-term COVID. Translation of the English version of the C19-YRS into the Thai language and testing it is essential for the psychometric evaluation of the severity of the long-term COVID syndrome prior to providing rehabilitation care for community members.

**Methods:**

Forward-and back-translations including cross-cultural aspects were conducted in order to develop a preliminary Thai version of that tool. Five experts evaluated the content validity of the tool and produced a highly valid index. A cross-sectional study was then conducted on a sample of 337 Thai community members recovering from COVID-19. Assessment of internal consistency and individual item analyses were also performed.

**Results:**

The content validity resulted in valid indices. The analyses showed that 14 items had acceptable internal consistency, based on the corrected item correlations. However, five symptom severity items and two functional ability items were deleted. The Cronbach’s alpha coefficient of the final C19-YRS was 0.723, indicating acceptable internal consistency and reliability of the survey instrument.

**Conclusions:**

This study indicated that the Thai C19-YRS tool had acceptable validity and reliability for the evaluation and testing of the psychometric variables in a Thai community population. The survey instrument also had acceptable validity and reliability for screening the symptoms and severity of long-term COVID. Further studies are warranted in order to standardize the various applications of this tool.

**Supplementary Information:**

The online version contains supplementary material available at 10.1186/s12889-023-15566-2.

## Background

The Coronavirus disease 2019 (COVID-19) pandemic began in late 2019, and the number of cases escalated rapidly. On January 22, 2020, the first case of COVID-19 was reported in Thailand. During the first outbreak (January-March, 2020), the number of affected individuals grew progressively; however, the first outbreak of this pandemic was relatively manageable in Thailand. The pandemic worsened however from mid-March 2020 onward. Because of a variation in the corona virus strain, the pandemic’s second wave spread throughout Thailand, followed by numerous waves of this viral infection [1].

Follow-up examinations of the symptoms in COVID-19 patients revealed that some cases developed difficulties in some systems after recovering from the acute phase. Coughs, trouble breathing, chest pain, perception disorders, and fatigue are the most prevalent symptoms [2]. This syndrome is termed the long-term COVID or the post-COVID-19 condition (4–12 weeks post COVID-19) by the National Institute for Health and Care Excellence (NICE) after the onset of the acute phase [2, 3].

Long-term COVID affects essentially all bodily systems, including respiratory, cardiovascular, blood, nervous, gastrointestinal, urinary, metabolic, musculoskeletal, cognitive, and integumentary systems [3–5]. The severity of the symptoms in each system varies widely depending on such factors as age, underlying conditions, pregnancy, obesity, lack of exercise, smoking, and the use of various drugs [6]. Long-term COVID requires follow-up care in order to assess the treatment efficacy and recovery. The NICE recommends that the rehabilitative approach for this condition be carried out by a multidisciplinary team (MDT); this will help adjust the therapeutic program based on the individual patient’s needs [2]. As part of such a program, determining the severity of the long-term COVID syndrome is of critical importance in planning and establishing optimal care.

The C19-YRS is a standardized tool to monitor the treatment and recovery process for long-term COVID. This tool is designed to screen the most common symptoms, as listed in Table 1. The scores generated from Table 1 are categorized into three levels of severity: mild, moderate, and severe [7].

**Table 1:** The original C19-YRS Items and Subscales

Subscales	C19-YRS Items
Symptoms	Dyspnea Laryngeal/respiratory complications Speech Swallowing Nutrition Fatigue Urinary incontinence, Bowel incontinence Pain/discomfort Cognition Anxiety Depression Post-traumatic stress disorder (PTSD)
Functional ability	Communication Self-care Movement Activities of daily living Occupational, and family/caregiver opinions
Overall health	Perception of overall health

The C19-YRS has been widely used to screen the symptoms and severity of long-term COVID in patients that have been discharged from the hospital and those recovering in typical European communities [8–14] and west Asian nations [15, 16]. Research into long-term COVID in Thailand has led to limited data; thus, further evidence to support long-term COVID management by MDT is warranted. Therefore, the current study was planned to translate the English C19-YRS into a Thai version, and then to test its psychometric properties for community members in Bangkok, Thailand.

## Methods

### Study design and participants

This cross-sectional study was conducted with a sample of 344 community members in Bangkok, Thailand. The inclusion criteria were as follows.

- Individuals aged over 20 years
- Those recovering from COVID-19
- Living in Ratchathewi District, Bangkok, Thailand

Based on a former study, the ideal sample size for the item analysis is 10 participants for every item [17]. Specifically to reduce the error rate, the recommended optimal ratio is 20:1 for the participants to items ratio [18]. In the current study, the sample size was estimated to be 210–420 for the 21-item instrument. The actual sample size was within the estimated range, and a researcher collected the data from subjects that met the inclusion criteria.

### Translation process

The researchers accessed the licensing authority at the University of Leeds, United Kingdom, for self-identification and requested permission to use the C19-YRS in Thailand. After obtaining approval, the research team began the translation process. They used a cross-cultural adaptation [19] as a guideline for translating the C19-YRS according to the approved recommendations. The original English version of the C19-YRS was forward-translated into the Thai language by two independent bilingual translators. One translator was an instructor with expertise in community health nursing and disaster (T1); the second translator was a physical therapist expert in cardiovascular and pulmonary rehabilitation (T2). A meeting was held to merge the drafts produced by the two translators for the purpose of forward-translation (T-12). Then, a second translator, with no healthcare background, back-translated the T-12 version into English while being blinded to the original version. This process was employed for a validity check and to determine the similarity of the content to the original version (T1).

Next, both the original and back-translated versions were reviewed in order to reach a consensus regarding the semantic and conceptual equivalence by an expert committee. The back-translated English draft was sent to its original developer following approval by the Leeds licensing authority in order to check the appropriateness of the cross-cultural equivalence. Using the content validity index, five expert individuals (two community health nursing specialists, two rehabilitation specialists, and one instrument development expert) evaluated the content validity of the C19-YRS preliminary translated Thai version (CVI). These experts were asked to rank each item on the basis of how appropriate and relevant the C19-YRS preliminary translated Thai version was. The CVI was 0.95, indicating that the contents were sufficiently valid and relevant [20].

The Thai version of the C19-YRS preliminary tool was pilot tested with 30 participants that had recovered from COVID-19 in Bangkok, and then was re-tested with a group of similar participants after two weeks. The correlation coefficient of the results over time was greater than 0.78, indicating good internal consistency [21]. Some modifications were made in order to make the translated Thai version easily understandable in the Thai context.

### Measures

The C19-YRS was developed by Sivan and MDT rehabilitation professionals in 2021 [7, 22]. The scale was used to monitor the long-term COVID and the post-COVID-19 syndrome. The C19-YRS consisted of 25 items, starting with two opening questions, followed by 15 questions focusing on the main symptoms. Five questions concentrated on functional abilities and social roles, one question was related to perceived health status, one question was linked to family perspectives, and finally, one question addressed other symptoms not mentioned in the other questions. The major symptoms (n = 15) consisted of the following.

- Breathlessness at rest, when dressing, or caused by walking up stairs
- Coughs or throat discomfort
- Changes in voice
- Difficulty swallowing
- Nutritional concern
- Fatigue
- Incontinence
- Pain or discomfort
- Difficulty with concentration
- Short-term memory
- Difficulty with planning
- Anxiety or depression
- PTSD

Further, the participants answered the following five *“Yes”* or *“No”* questions related to functional abilities and social roles: (a) communication, (b) mobility, (c) personal care, (d) activities of daily living, and (e) social roles. Answering “***Yes***” indicated that the participants experienced an adverse effect of COVID-19 regarding those symptoms and functional impairments. The participants were asked to rate the severity of their symptoms on a scale of zero to 10, and they were also asked to compare their symptoms concerning to how they felt prior to having COVID-19.

Regarding interpretation, the C19-YRS was divided into three subscales, consisting of symptom severity (15 items), functional ability (five items), and overall health (one item). The score for each symptom was divided into the three categories of mild (0 to 2), moderate (3 to 5), and severe (6 or higher). The total score for the symptom severity subscale ranged from zero to 150. The total score representing functional ability ranged from zero to 50, and the score for overall health ranged from zero to 10.

### Data collection

The data collection process started after obtaining approval from the Institutional Review Board (IRB) Committee on Human Research. The data were collected between November 2021 and May 2022. The researcher contacted community leaders in Bangkok, described the study objectives, and sought their cooperation in securing contact information on potential participants. Two data collection methods were used, depending on the participants’ ability to use and access the Internet. In some cases, participants accessed the online questionnaire, and the researcher or coordinators provided a QR code to access it. The participants took approximately 15 min to answer the questionnaire by themselves. For the participants that were unable to access the online questionnaire, the researcher or study coordinators collected the data using a paper-based questionnaire.

### Statistical analysis

The data were analyzed using the Statistical Package for Social Sciences (SPSS) for Windows, version 21. Missing data were excluded before the analysis. An overview of the participants’ characteristics was presented using descriptive statistics. Cronbach’s alpha coefficient was used to assess the internal consistency of the items, and item analyses were performed in order to assess the reliability of the instrument according to Ferketich’s study [21].

## Results

### Participants’ characteristics

Among the 337 community members, 59 (17.5%) were over 60 years of age, and over half of the participants were female (53.7%). Some of the participants were from Myanmar, Cambodia, and Laos, and all of the participants were able to communicate in the Thai language. The majority of participants had completed secondary education (40.9%), and approximately one in five participants were unemployed (23.1%). The majority of the participants (n = 23; 88.1%) had minor symptoms during their COVID-19 infection, and were included in the green group category [23]. The participants’ characteristics was shown in Supplementary Table 1. Clinical presentation and functional disability of long-term COVID was presented in Supplementary Table 2.

### Items analyses

Cronbach’s alpha coefficient of the preliminary C19-YRS instrument in this study with 21 items was 0.702. A similar coefficient for the symptom severity subscale (n = 15 items) was 0.723. This finding indicated good internal consistency and reliability for the instrument. Further, the coefficient for the functional ability subscale (n = 5 items) was 0.588, indicating acceptable internal consistency and reliability. See Table 2.

**Table 2:** Summary of item analysis of the C19-YRS Thai version (N = 337)

Item analysis	Criteria	Results	Interpretations
Corrected item–total correlations			
Symptom severity	0.30–0.70	0.320 to 0.557 (10 items)	Acceptable internal consistency
		0.143 (breathlessness at dressing) 0.036 (swallowing difficulty) 0.063 (continence) 0.246 (anxiety) 0.164 (PTSD)	Not acceptable internal consistency
Functional ability		0.495 to 0.537 (three items)	Acceptable internal consistency
		0.284 (personal care) 0.143 (social role)	Not acceptable internal consistency
**Item–subscale correlations**			
Symptom severity	≥ 0.5	0.444–0.750 (10 items)	Acceptable internal consistency
Functional ability		0.427–0.957(three items)	
**Subscale–subscale correlation coefficients**			
Symptom severity-Functional ability	0.40–0.65	0.594	Acceptable
**Subscale–total correlation coefficients**			
Symptom severity-total score	0.55–0.88	0.906	Higher than acceptable criteria
Functional ability-total score		0.741	Acceptable

### Corrected item-total correlations

An earlier study recommended that the corrected item correlation should be between 0.30 and 0.70 [21]. In this study, the correlations for the preliminary C19-YRS symptoms severity ranged consistently between 0.320 and 0.557. Five items did not meet the criteria, including shortness of breath when dressing (0.143), swallowing difficulty (0.036), incontinence (0.063), anxiety (0.246), and PTSD (0.164). The corrected item correlations for the preliminary C19-YRS functional ability ranged between 0.495 and 0.537. As shown in Table 2, two items did not meet the criteria, which were deleted before further analyses. These items were personal care (0.284) and social roles (0.143).

### Item-subscale correlations

The correlations for items related to symptom severity correctly ranged between 0.444 and 0.750. One item did not meet the criteria for “breathlessness in walking up stairs” (r = 0.444); however, it was deemed acceptable because the correlation coefficient was close to 0.5. The items correlations for the functional ability subscale ranged between 0.427 and 0.957. One item did not meet the criteria: “communication” (r = 0.427); however, this item was deemed acceptable because its correlation coefficient approached 0.5, which demonstrated acceptable internal consistency. See Tables 2 and 3.

**Table 3:** The correlation coefficients for subscale–subscale and subscale–total scale of the C19-YRS Thai version (N = 337)

Pearson’s correlation	Symptom severity	Functional ability	Overall health	Total score
Symptom severity	1			
Functional ability	0.594*	1		
Overall health	−0.055	−0.101	1	
Total score	0.906*	0.741*	0.267*	1

*p-value < 0.01

### Subscale-subscale correlation coefficients

The subscale-subscale correlation coefficients ranged between 0.40 and 0.65 [21]. The correlation coefficient for the symptom severity and functional ability scores was acceptable at 0.594. In this category, the correlation coefficient should range between 0.55 and 0.88 [21].

### Subscale-total correlation coefficients

The correlation coefficient between symptom severity and the total score was 0.906, which meets the acceptable criterion. The correlation coefficient between functional ability and the total score was 0.741, which was acceptable, as shown in Tables 2 and 3.

In summary, the item analysis showed that five items in the symptom severity and three in the functional ability categories did not have acceptable internal consistency. Thus, these items were excluded from the instrument before re-analysis of the final C19-YRS version. A summary of the item analyses is presented in Table 2.

Cronbach’s alpha coefficient of the final C19-YRS was re-analyzed after deletion of the unacceptable items. Cronbach’s alpha coefficient of the final C19-YRS (14 items) was 0.723, and Cronbach’s alpha coefficient for the symptom severity subscale (10 items) was 0.794, indicating good internal consistency and reliability. Furthermore, Cronbach’s alpha coefficient for the functional ability subscale (three items) was 0.699, indicating acceptable internal consistency and reliability, as shown in Table 4.

**Table 4:** The final C19-YRS Thai version 14 items (N = 337)

Subscale	Symptom severity	Functional disability	Overall health
Scale range	0–100	0–30	0–10
Score range	0–64	0–20	0–10
Mean (SD)	2.089 (5.421)	0.528 (2.642)	8.047 (2.580)
Number of items	10	3	1
Corrected item–total correlations	0.320–0.557	0.495–0.537	**-**
Item–subscale correlations	0.444–0.750	0.427–0.957	-
Cronbach’s alpha coefficient	0.794	0.699	-

## Discussion

This is the first study to test the C19-YRS among Thai community members in Bangkok, Thailand. The C19-YRS Thai version showed acceptable reliability, with a Cronbach’s alpha coefficient of 0.723. This value was lower than that of the original C19-YRS version (α = 0.891) [24].

The items related to swallowing difficulty and incontinence did not demonstrate acceptable internal consistency in the Thai version of the C19-YRS, which was similar to the results of the original version (24). As only two participants reported each of these symptoms (as shown in Supplementary Table 2), and because there is little evidence to support their association with long-term COVID, they were considered to be excluded from the Thai version. The current findings support the modified version of the C19-YRS [11], which includes these two symptoms in the “other symptoms” section.

The item of breathlessness at dressing demonstrated unacceptable internal consistency. The participants may have been unable to distinguish between their perceptions of breathlessness while dressing and breathlessness while at rest. Breathlessness may not need to be divided into subcategories when applying the tool in mild cases.

Symptoms of anxiety (28.5%) and PTSD (18.2%) were also shown to have unacceptable internal consistency in this study. As the Rasch model analysis demonstrated the local dependence of these items when scored separately in the original version of the C19-YRS, they have been grouped in the “psychological symptoms” section in the modified version [11].

Regarding symptom severity and functional ability, the mean scores of the final C19-YRS Thai version were lower than those reported in the original version, while the mean score for overall health was higher than that for the original version [24]. This may be due to the fact that most of the participants in the current study experienced only mild COVID-19 infection symptoms, and were classified in the green group category, as a community-based approach was implemented in Thailand for mild cases or the green group category [23, 25]. Thus, the prevalence of symptoms and functional impairments was lower in patients that were not admitted to the intensive care unit (ICU) than those who were admitted to the ICU. The former patients were more likely to have a better quality of life than others in the study population [9].

### Limitation of the study

Some of the study’s limitations involved in the translation process should be considered. According to the cross-cultural adaptation process, one of the forward translators should have no medical background in order to reflect the language used by the general population. However, the preliminary Thai version was pilot tested and revised to ensure it adequately understandable to the community members.

### Recommendations for future studies

In future research, we plan to contact COVID-19 survivors who have received outpatient care in order to compare the circumstances surrounding their symptoms and their functional ability. Moreover, the use of multidisciplinary care models for long-term COVID management should be encouraged, serving as a link between hospitals and community healthcare facilities.

## Conclusions

This study provides a preliminary standardization of the C19-YRS Thai version. The Thai version of C19-YRS demonstrated acceptable validity and reliability, with majority of the findings consistent with the modified version of the tool. However, breathlessness at dressing was not found to be a significant symptom in the Thai context. The Thai version of the C19-YRS may be useful for assessing rehabilitation needs in community settings in Thailand.

## Electronic supplementary material

Below is the link to the electronic supplementary material.

Supplementary Material 1

Supplementary Material 2

Supplementary Material 3

Supplementary Material 4

## Data Availability

The datasets used and/or analysed during the current study are available from the corresponding author on reasonable request.

## Abbreviations

C19-YRS: COVID-19 Yorkshire Rehabilitation Scale
NICE: National Institute for Health and Care Excellence
MDT: multidisciplinary team
PTSD: post-traumatic stress disorder
ICU: intensive care unit
