# Supplementary material for: The Thai version of the COVID-19 Yorkshire Rehabilitation Scale: a valid instrument for the psychometric assessment of the community members in Bangkok, Thailand
Source: BMC Public Health. 2023 Apr 11;23:663. doi: 10.1186/s12889-023-15566-2 (PMC10088103; doi:10.1186/s12889-023-15566-2)
Supplement: Supplementary file 1 — Supplementary Material 1 [file 12889_2023_15566_MOESM1_ESM.docx]

**Supplementary table 1: Characteristics of participants (N = 337)**

| **Characteristics** | **N** | **Percentage** |
| --- | --- | --- |
| **Age (years)** mean: 45.94; SD: 15.61 |  |  |
| 21–60 | 278 | 82.5 |
| Over 60 | 59 | 17.5 |
| **Gender** |  |  |
| Female | 181 | 53.7 |
| Male | 156 | 46.3 |
| **Ethnicity** |  |  |
| Thai | 324 | 96.1 |
| Myanmar | 6 | 1.8 |
| Cambodia | 3 | 0.9 |
| Lao | 4 | 1.2 |
| **Educational level** |  |  |
| No education | 22 | 6.5 |
| Primary education | 131 | 38.9 |
| Secondary education | 138 | 40.9 |
| Bachelor’s degree | 43 | 12.8 |
| Master’s degree | 3 | 0.9 |
| **Occupation** |  |  |
| Unemployment | 78 | 23.1 |
| Employment | 100 | 29.7 |
| Sales | 67 | 19.9 |
| Governor | 6 | 1.8 |
| State enterprise employee | 7 | 2.1 |
| Private company employees | 63 | 18.7 |
| Personal business | 11 | 3.3 |
| Student | 5 | 1.5 |
| **Symptom categories during COVID-19 infection** |  |  |
| Green | 297 | 88.1 |
| Yellow | 29 | 8.6 |
| Red | 11 | 3.3 |
| **Duration of long COVID (weeks)** mean: 26.00; SD: 9.80 |  |  |
