# Supplementary material for: The Thai version of the COVID-19 Yorkshire Rehabilitation Scale: a valid instrument for the psychometric assessment of the community members in Bangkok, Thailand
Source: BMC Public Health. 2023 Apr 11;23:663. doi: 10.1186/s12889-023-15566-2 (PMC10088103; doi:10.1186/s12889-023-15566-2)
Supplement: Supplementary file 2 — Supplementary Material 2 [file 12889_2023_15566_MOESM2_ESM.docx]

**Supplementary table 2: Clinical presentation and functional disability of long-term COVID (N = 337)**

| **Symptoms** | **N** | **Percentage** |
| --- | --- | --- |
| Breathlessness at rest | 15 | 4.5 |
| Breathlessness at dressing | 16 | 4.7 |
| Breathlessness on walking up a flight of stairs | 45 | 13.4 |
| Cough or throat sensitivity | 19 | 5.6 |
| Voice change | 6 | 1.8 |
| Swallowing difficulty | 2 | 0.6 |
| Nutritional concern | 16 | 4.7 |
| Fatigue | 88 | 26.1 |
| Incontinence | 2 | 0.6 |
| Pain | 27 | 8.0 |
| Concentrating | 7 | 2.1 |
| Short term memory | 16 | 4.7 |
| Anxiety | 96 | 28.5 |
| Depression | 7 | 2.1 |
| Post-traumatic stress disorder (PTSD screen) | 62 | 18.2 |
| **Functional disability** |  |  |
| Communication | 5 | 1.5 |
| Mobility | 18 | 5.3 |
| Personal care | 6 | 1.8 |
| Usual activities of daily living | 16 | 4.7 |
| Social roles | 69 | 20.5 |
