# Supplementary material for: The Thai version of the COVID-19 Yorkshire Rehabilitation Scale: a valid instrument for the psychometric assessment of the community members in Bangkok, Thailand
Source: BMC Public Health. 2023 Apr 11;23:663. doi: 10.1186/s12889-023-15566-2 (PMC10088103; doi:10.1186/s12889-023-15566-2)
Supplement: Supplementary file 4 — Supplementary Material 4 [file 12889_2023_15566_MOESM4_ESM.pdf]

```
RELIABILITY
  /VARIABLES=dyspnea_rest_now_severity dyspnea_dressing_now_severity dyspnea_s
tair_now_severity larynx_prob_serverity voice_impact_severity swallowing_impac
t_severity nutrition_impact_severity mobility_pc_now_severity fatigue_now_seve
rity
personalcare_now_level Continenc ADL_now_level painnow_severity concentration
short_memory communication_now_level anxiety_now_level depression_now_level PT
SD overallhealth_now_level socialrole
  /SCALE('ALL VARIABLES') ALL
  /MODEL=ALPHA
  /STATISTICS=DESCRIPTIVE SCALE
  /SUMMARY=TOTAL CORR.
```

Reliability

Notes

Output Created		28-Nov-2022 16:14:45
Comments		
Input	Data	C:\Users\CNMI.DESKTOP-R9ODJFL\Desktop\YRS for MAI.sav
	Active Dataset	DataSet1
	Filter	<none>
	Weight	<none>
	Split File	<none>
	N of Rows in Working Data File	337
	Matrix Input	
Missing Value Handling	Definition of Missing	User-defined missing values are treated as missing.
	Cases Used	Statistics are based on all cases with valid data for all variables in the procedure.

Notes

Syntax		RELIABILITY<br>/VARIABLES=dyspnea_rest_now_severity dyspnea_dressing_now_severity dyspnea_stair_now_severity larynx_prob_serverity voice_impact_severity swallowing_impact_severity nutrition_impact_severity mobility_pc_now_severity fatigue_now_severity personalcare_now_level Continenc ADL_now_level painnow_severity concentration short_memory communication_now_level anxiety_now_level depression_now_level PTSD overallhealth_now_level socialrole<br>/SCALE('ALL VARIABLES') ALL<br>/MODEL=ALPHA<br>/STATISTICS=DESCRIPTIVE SCALE<br>/SUMMARY=TOTAL CORR.
Resources	Processor Time	00:00:00.000
	Elapsed Time	00:00:00.006

```
[DataSet1] C:\Users\CNMI.DESKTOP-R9ODJFL\Desktop\YRS for MAI.sav
```

Scale: ALL VARIABLES

Case Processing Summary

		N	%
Cases	Valid	337	100.0
	Excluded[a]	0	.0
	Total	337	100.0

a. Listwise deletion based on all variables in the procedure.

Reliability Statistics

Cronbach's Alpha	Cronbach's Alpha Based on Standardized Items	N of Items
.702	.816	21

Item Statistics

	Mean	Std. Deviation	N
dyspnea_rest_now_ severity	.0920	.51220	337
dyspnea_dressing_now_ severity	.1246	.78446	337
dyspnea_stair_now_ severity	.3769	1.25017	337
larynx_prob_serverity	.1335	.74998	337
voice_impact_severity	.0475	.44064	337
swallowing_impact_ severity	.0119	.15384	337
nutrition_impact_severity	.1009	.53611	337
mobility_pc_now_severity	.2522	1.32238	337
fatigue_now_severity	.7181	1.56622	337
personalcare_now_level	.1217	1.06068	337
Continenc	.0148	.19614	337
ADL_now_level	.2255	1.28042	337
painnow_severity	.2047	.91429	337
concentration	.0950	.68782	337
short_memory	.1958	.93075	337
communication_now_level	.0504	.58285	337
anxiety_now_level	1.2878	2.48273	337
depression_now_level	.1246	.94926	337
PTSD	.5401	1.16203	337
overallhealth_now_level	8.0475	2.58040	337
socialrole	.7240	1.93598	337

Summary Item Statistics

	Mean	Minimum	Maximum	Range	Maximum / Minimum	Variance
Inter-Item Correlations	.175	-.395	.839	1.233	-2.125	.046

Summary Item Statistics

	N of Items
Inter-Item Correlations	21

Item-Total Statistics

	Scale Mean if Item Deleted	Scale Variance if Item Deleted	Corrected Item-Total Correlation
dyspnea_rest_now_severity	13.3976	91.324	.451
dyspnea_dressing_now_severity	13.3650	93.215	.143
dyspnea_stair_now_severity	13.1128	86.975	.320
larynx_prob_serverity	13.3561	90.016	.381
voice_impact_severity	13.4421	91.438	.518
swallowing_impact_severity	13.4777	95.869	.036
nutrition_impact_severity	13.3887	90.310	.530
mobility_pc_now_severity	13.2374	82.366	.495
fatigue_now_severity	12.7715	78.760	.532
personalcare_now_level	13.3680	89.186	.284
Continenc	13.4748	95.720	.063
ADL_now_level	13.2641	81.921	.537
painnow_severity	13.2849	85.734	.557
concentration	13.3947	88.823	.517
short_memory	13.2938	85.803	.541
communication_now_level	13.4392	89.973	.514
anxiety_now_level	12.2018	78.977	.246
depression_now_level	13.3650	87.197	.446
PTSD	12.9496	91.018	.164
overallhealth_now_level	5.4421	94.158	-.096
socialrole	12.7656	87.079	.143

Item-Total Statistics

	Squared Multiple Correlation	Cronbach's Alpha if Item Deleted
dyspnea_rest_now_severity	.695	.689
dyspnea_dressing_now_severity	.671	.700
dyspnea_stair_now_severity	.668	.686
larynx_prob_serverity	.598	.687
voice_impact_severity	.741	.688
swallowing_impact_severity	.128	.703
nutrition_impact_severity	.518	.685
mobility_pc_now_severity	.887	.668
fatigue_now_severity	.465	.660
personalcare_now_level	.908	.690
Continenc	.362	.703
ADL_now_level	.910	.664
painnow_severity	.660	.672
concentration	.597	.681
short_memory	.606	.673
communication_now_level	.802	.684
anxiety_now_level	.109	.710
depression_now_level	.478	.679
PTSD	.391	.700
overallhealth_now_level	.238	.771
socialrole	.230	.713

Scale Statistics

Mean	Variance	Std. Deviation	N of Items
13.4896	96.001	9.79799	21

```
RELIABILITY
  /VARIABLES=dyspnea_rest_now_severity dyspnea_dressing_now_severity dyspnea_s
tair_now_severity larynx_prob_serverity voice_impact_severity swallowing_impac
t_severity nutrition_impact_severity fatigue_now_severity Continenc painnow_se
verity concentration
short_memory anxiety_now_level depression_now_level PTSD
  /SCALE('ALL VARIABLES') ALL
  /MODEL=ALPHA
  /STATISTICS=DESCRIPTIVE SCALE
```

```
  /SUMMARY=TOTAL CORR.
```

Reliability symptom subscale

Notes

Output Created		28-Nov-2022 16:16:41
Comments		
Input	Data	C:\Users\CNMI.DESKTOP-R9ODJFL\Desktop\YRS for MAI.sav
	Active Dataset	DataSet1
	Filter	<none>
	Weight	<none>
	Split File	<none>
	N of Rows in Working Data File	337
	Matrix Input	
Missing Value Handling	Definition of Missing	User-defined missing values are treated as missing.
	Cases Used	Statistics are based on all cases with valid data for all variables in the procedure.
Syntax		RELIABILITY /VARIABLES=dyspnea_rest_now_severity dyspnea_dressing_now_severity dyspnea_stair_now_severity larynx_prob_serverity voice_impact_severity swallowing_impact_severity nutrition_impact_severity fatigue_now_severity Continenc painnow_severity concentration short_memory anxiety_now_level depression_now_level PTSD /SCALE('ALL VARIABLES') ALL /MODEL=ALPHA /STATISTICS=DESCRIPTIVE SCALE /SUMMARY=TOTAL CORR.
Resources	Processor Time	00:00:00.000
	Elapsed Time	00:00:00.003

```
[DataSet1] C:\Users\CNMI.DESKTOP-R9ODJFL\Desktop\YRS for MAI.sav
```

Scale: ALL VARIABLES

Case Processing Summary

		N	%
Cases	Valid	337	100.0
	Excluded[a]	0	.0
	Total	337	100.0

a. Listwise deletion based on all variables in the procedure.

Reliability Statistics

Cronbach's Alpha	Cronbach's Alpha Based on Standardized Items	N of Items
.723	.796	15

Item Statistics

	Mean	Std. Deviation	N
dyspnea_rest_now_ severity	.0920	.51220	337
dyspnea_dressing_now_ severity	.1246	.78446	337
dyspnea_stair_now_ severity	.3769	1.25017	337
larynx_prob_serverity	.1335	.74998	337
voice_impact_severity	.0475	.44064	337
swallowing_impact_ severity	.0119	.15384	337
nutrition_impact_severity	.1009	.53611	337
fatigue_now_severity	.7181	1.56622	337
Continenc	.0148	.19614	337
painnow_severity	.2047	.91429	337
concentration	.0950	.68782	337
short_memory	.1958	.93075	337
anxiety_now_level	1.2878	2.48273	337
depression_now_level	.1246	.94926	337
PTSD	.5401	1.16203	337

Summary Item Statistics

	Mean	Minimum	Maximum	Range	Maximum / Minimum	Variance
Inter-Item Correlations	.206	-.028	.668	.697	-23.551	.034

Summary Item Statistics

	N of Items
Inter-Item Correlations	15

Item-Total Statistics

	Scale Mean if Item Deleted	Scale Variance if Item Deleted	Corrected Item-Total Correlation
dyspnea_rest_now_ severity	3.9763	47.059	.516
dyspnea_dressing_now_ severity	3.9436	48.750	.145
dyspnea_stair_now_ severity	3.6914	44.012	.324
larynx_prob_serverity	3.9347	45.639	.469
voice_impact_severity	4.0208	47.360	.560
swallowing_impact_ severity	4.0564	50.821	.048
nutrition_impact_severity	3.9674	47.567	.419
fatigue_now_severity	3.3501	38.794	.497
Continenc	4.0534	50.533	.136
painnow_severity	3.8635	43.047	.589
concentration	3.9733	45.407	.547
short_memory	3.8724	43.618	.526
anxiety_now_level	2.7804	37.636	.235
depression_now_level	3.9436	43.690	.507
PTSD	3.5282	45.018	.294

Item-Total Statistics

	Squared Multiple Correlation	Cronbach's Alpha if Item Deleted
dyspnea_rest_now_ severity	.644	.703
dyspnea_dressing_now_ severity	.572	.724
dyspnea_stair_now_ severity	.605	.709
larynx_prob_serverity	.538	.699
voice_impact_severity	.609	.704
swallowing_impact_ severity	.119	.726
nutrition_impact_severity	.391	.708
fatigue_now_severity	.448	.684
Continenc	.296	.724
painnow_severity	.609	.683
concentration	.492	.695
short_memory	.527	.689
anxiety_now_level	.092	.779
depression_now_level	.397	.690
PTSD	.245	.712

Scale Statistics

Mean	Variance	Std. Deviation	N of Items
4.0682	50.951	7.13797	15

```
RELIABILITY
  /VARIABLES=mobility_pc_now_severity personalcare_now_level ADL_now_level com
munication_now_level socialrole
  /SCALE('ALL VARIABLES') ALL
  /MODEL=ALPHA
  /STATISTICS=DESCRIPTIVE SCALE
  /SUMMARY=TOTAL CORR.
```

Reliability functional ability subscale

Notes

Output Created		28-Nov-2022 16:17:56
Comments		
Input	Data	C:\Users\CNMI.DESKTOP-R9ODJFL\Desktop\YRS for MAI.sav
	Active Dataset	DataSet1
	Filter	<none>
	Weight	<none>
	Split File	<none>
	N of Rows in Working Data File	337
	Matrix Input	
Missing Value Handling	Definition of Missing	User-defined missing values are treated as missing.
	Cases Used	Statistics are based on all cases with valid data for all variables in the procedure.
Syntax		RELIABILITY /VARIABLES=mobility_pc_now_severity personalcare_now_level ADL_now_level communication_now_level socialrole /SCALE('ALL VARIABLES') ALL /MODEL=ALPHA /STATISTICS=DESCRIPTIVE SCALE /SUMMARY=TOTAL CORR.
Resources	Processor Time	00:00:00.000
	Elapsed Time	00:00:00.003

```
[DataSet1] C:\Users\CNMI.DESKTOP-R9ODJFL\Desktop\YRS for MAI.sav
```

Scale: ALL VARIABLES

Case Processing Summary

		N	%
Cases	Valid	337	100.0
	Excluded[a]	0	.0
	Total	337	100.0

a. Listwise deletion based on all variables in the procedure.

Reliability Statistics

Cronbach's Alpha	Cronbach's Alpha Based on Standardized Items	N of Items
.588	.659	5

Item Statistics

	Mean	Std. Deviation	N
mobility_pc_now_severity	.252	1.3224	337
personalcare_now_level	.122	1.0607	337
ADL_now_level	.226	1.2804	337
communication_now_level	.050	.5828	337
socialrole	.724	1.9360	337

Summary Item Statistics

	Mean	Minimum	Maximum	Range	Maximum / Minimum	Variance
Inter-Item Correlations	.279	-.052	.839	.890	-16.237	.142

Summary Item Statistics

	N of Items
Inter-Item Correlations	5

Item-Total Statistics

	Scale Mean if Item Deleted	Scale Variance if Item Deleted	Corrected Item-Total Correlation
mobility_pc_now_severity	1.122	9.220	.656
personalcare_now_level	1.252	10.701	.635
ADL_now_level	1.148	9.377	.665
communication_now_level	1.323	15.333	.123
socialrole	.650	12.675	-.014

Item-Total Statistics

	Squared Multiple Correlation	Cronbach's Alpha if Item Deleted
mobility_pc_now_severity	.714	.342
personalcare_now_level	.843	.402
ADL_now_level	.871	.343
communication_now_level	.527	.615
socialrole	.033	.823

Scale Statistics

Mean	Variance	Std. Deviation	N of Items
1.374	16.235	4.0292	5

Notes

Output Created		28-Nov-2022 16:23:19
Comments		
Input	Data	C:\Users\CNMI.DESKTOP-R9ODJFL\Desktop\YRS for MAI.sav
	Active Dataset	DataSet1
	Filter	<none>
	Weight	<none>
	Split File	<none>
	N of Rows in Working Data File	337
Missing Value Handling	Definition of Missing	User-defined missing values are treated as missing.
	Cases Used	Statistics for each pair of variables are based on all the cases with valid data for that pair.
Syntax		CORRELATIONS /VARIABLES=Symptom dyspnea_rest_now_severity dyspnea_dressing_now_severity dyspnea_stair_now_severity larynx_prob_serverity voice_impact_severity swallowing_impact_severity nutrition_impact_severity fatigue_now_severity Continenc painnow_severity concentration short_memory anxiety_now_level depression_now_level PTSD /PRINT=TWOTAIL NOSIG /MISSING=PAIRWISE.
Resources	Processor Time	00:00:00.000
	Elapsed Time	00:00:00.010

```
CORRELATIONS
  /VARIABLES=dyspnea_rest_now_severity dyspnea_stair_now_severity larynx_prob_
serverity voice_impact_severity nutrition_impact_severity fatigue_now_severity
 painnow_severity concentration short_memory depression_now_level symptom10ite
ms
  /PRINT=TWOTAIL NOSIG
  /MISSING=PAIRWISE.
```

Correlations

item-subscale correlation (symptom severity)

Notes

Output Created		28-Nov-2022 16:25:38
Comments		
Input	Data	C:\Users\CNMI.DESKTOP-R9ODJFL\Desktop\YRS for MAI.sav
	Active Dataset	DataSet1
	Filter	<none>
	Weight	<none>
	Split File	<none>
	N of Rows in Working Data File	337
Missing Value Handling	Definition of Missing	User-defined missing values are treated as missing.
	Cases Used	Statistics for each pair of variables are based on all the cases with valid data for that pair.
Syntax		CORRELATIONS /VARIABLES=dyspnea_rest_now_severity dyspnea_stair_now_severity larynx_prob_serverity voice_impact_severity nutrition_impact_severity fatigue_now_severity painnow_severity concentration short_memory depression_now_level symptom10items /PRINT=TWOTAIL NOSIG /MISSING=PAIRWISE.
Resources	Processor Time	00:00:00.016
	Elapsed Time	00:00:00.007

```
[DataSet1] C:\Users\CNMI.DESKTOP-R9ODJFL\Desktop\YRS for MAI.sav
```

Correlations

		dyspnea_rest_now_severity	dyspnea_stair_now_severity	larynx_prob_serverity
dyspnea_rest_now_severity	Pearson Correlation	1	.211**	.588**
	Sig. (2-tailed)		.000	.000
	N	337	337	337
dyspnea_stair_now_severity	Pearson Correlation	.211**	1	.060
	Sig. (2-tailed)	.000		.269
	N	337	337	337
larynx_prob_serverity	Pearson Correlation	.588**	.060	1
	Sig. (2-tailed)	.000	.269	
	N	337	337	337
voice_impact_severity	Pearson Correlation	.587**	.027	.611**
	Sig. (2-tailed)	.000	.623	.000
	N	337	337	337
nutrition_impact_severity	Pearson Correlation	.118*	.183**	.092
	Sig. (2-tailed)	.031	.001	.091
	N	337	337	337
fatigue_now_severity	Pearson Correlation	.262**	.181**	.298**
	Sig. (2-tailed)	.000	.001	.000
	N	337	337	337
painnow_severity	Pearson Correlation	.659**	.203**	.524**
	Sig. (2-tailed)	.000	.000	.000
	N	337	337	337
concentration	Pearson Correlation	.229**	.162**	.350**
	Sig. (2-tailed)	.000	.003	.000
	N	337	337	337
short_memory	Pearson Correlation	.249**	.274**	.223**
	Sig. (2-tailed)	.000	.000	.000
	N	337	337	337
depression_now_level	Pearson Correlation	.344**	.061	.403**
	Sig. (2-tailed)	.000	.267	.000
	N	337	337	337
symptom10items	Pearson Correlation	.603**	.444**	.594**
	Sig. (2-tailed)	.000	.000	.000
	N	337	337	337

**. Correlation is significant at the 0.01 level (2-tailed).

*. Correlation is significant at the 0.05 level (2-tailed).

Correlations

		voice_impact_severity	nutrition_impact_severity	fatigue_now_severity
dyspnea_rest_now_severity	Pearson Correlation	.587**	.118*	.262**
	Sig. (2-tailed)	.000	.031	.000
	N	337	337	337
dyspnea_stair_now_severity	Pearson Correlation	.027	.183**	.181**
	Sig. (2-tailed)	.623	.001	.001
	N	337	337	337
larynx_prob_serverity	Pearson Correlation	.611**	.092	.298**
	Sig. (2-tailed)	.000	.091	.000
	N	337	337	337
voice_impact_severity	Pearson Correlation	1	.181**	.334**
	Sig. (2-tailed)		.001	.000
	N	337	337	337
nutrition_impact_severity	Pearson Correlation	.181**	1	.520**
	Sig. (2-tailed)	.001		.000
	N	337	337	337
fatigue_now_severity	Pearson Correlation	.334**	.520**	1
	Sig. (2-tailed)	.000	.000	
	N	337	337	337
painnow_severity	Pearson Correlation	.582**	.328**	.462**
	Sig. (2-tailed)	.000	.000	.000
	N	337	337	337
concentration	Pearson Correlation	.427**	.353**	.453**
	Sig. (2-tailed)	.000	.000	.000
	N	337	337	337
short_memory	Pearson Correlation	.362**	.467**	.401**
	Sig. (2-tailed)	.000	.000	.000
	N	337	337	337
depression_now_level	Pearson Correlation	.484**	.192**	.338**
	Sig. (2-tailed)	.000	.000	.000
	N	337	337	337
symptom10items	Pearson Correlation	.641**	.544**	.739**
	Sig. (2-tailed)	.000	.000	.000
	N	337	337	337

**. Correlation is significant at the 0.01 level (2-tailed).

*. Correlation is significant at the 0.05 level (2-tailed).

Correlations

		painnow_ severity	concentration	short_memory
dyspnea_rest_now_ severity	Pearson Correlation	.659**	.229**	.249**
	Sig. (2-tailed)	.000	.000	.000
	N	337	337	337
dyspnea_stair_now_ severity	Pearson Correlation	.203**	.162**	.274**
	Sig. (2-tailed)	.000	.003	.000
	N	337	337	337
larynx_prob_serverity	Pearson Correlation	.524**	.350**	.223**
	Sig. (2-tailed)	.000	.000	.000
	N	337	337	337
voice_impact_severity	Pearson Correlation	.582**	.427**	.362**
	Sig. (2-tailed)	.000	.000	.000
	N	337	337	337
nutrition_impact_severity	Pearson Correlation	.328**	.353**	.467**
	Sig. (2-tailed)	.000	.000	.000
	N	337	337	337
fatigue_now_severity	Pearson Correlation	.462**	.453**	.401**
	Sig. (2-tailed)	.000	.000	.000
	N	337	337	337
painnow_severity	Pearson Correlation	1	.310**	.355**
	Sig. (2-tailed)		.000	.000
	N	337	337	337
concentration	Pearson Correlation	.310**	1	.575**
	Sig. (2-tailed)	.000		.000
	N	337	337	337
short_memory	Pearson Correlation	.355**	.575**	1
	Sig. (2-tailed)	.000	.000	
	N	337	337	337
depression_now_level	Pearson Correlation	.492**	.429**	.393**
	Sig. (2-tailed)	.000	.000	.000
	N	337	337	337
symptom10items	Pearson Correlation	.750**	.661**	.682**
	Sig. (2-tailed)	.000	.000	.000
	N	337	337	337

**. Correlation is significant at the 0.01 level (2-tailed).

Correlations

		depression_now_level	symptom10items
dyspnea_rest_now_severity	Pearson Correlation	.344**	.603**
	Sig. (2-tailed)	.000	.000
	N	337	337
dyspnea_stair_now_severity	Pearson Correlation	.061	.444**
	Sig. (2-tailed)	.267	.000
	N	337	337
larynx_prob_serverity	Pearson Correlation	.403**	.594**
	Sig. (2-tailed)	.000	.000
	N	337	337
voice_impact_severity	Pearson Correlation	.484**	.641**
	Sig. (2-tailed)	.000	.000
	N	337	337
nutrition_impact_severity	Pearson Correlation	.192**	.544**
	Sig. (2-tailed)	.000	.000
	N	337	337
fatigue_now_severity	Pearson Correlation	.338**	.739**
	Sig. (2-tailed)	.000	.000
	N	337	337
painnow_severity	Pearson Correlation	.492**	.750**
	Sig. (2-tailed)	.000	.000
	N	337	337
concentration	Pearson Correlation	.429**	.661**
	Sig. (2-tailed)	.000	.000
	N	337	337
short_memory	Pearson Correlation	.393**	.682**
	Sig. (2-tailed)	.000	.000
	N	337	337
depression_now_level	Pearson Correlation	1	.638**
	Sig. (2-tailed)		.000
	N	337	337
symptom10items	Pearson Correlation	.638**	1
	Sig. (2-tailed)	.000	
	N	337	337

**. Correlation is significant at the 0.01 level (2-tailed).

CORRELATIONS

```
  /VARIABLES=mobility_pc_now_severity ADL_now_level communication_now_level fu
nctional3items
  /PRINT=TWOTAIL NOSIG
  /MISSING=PAIRWISE.
```

Correlations

item-subscale correlation (functional ability)

Notes

Output Created		28-Nov-2022 16:28:49
Comments		
Input	Data	C:\Users\CNMI.DESKTOP-R9ODJFL\Desktop\YRS for MAI.sav
	Active Dataset	DataSet1
	Filter	<none>
	Weight	<none>
	Split File	<none>
	N of Rows in Working Data File	337
Missing Value Handling	Definition of Missing	User-defined missing values are treated as missing.
	Cases Used	Statistics for each pair of variables are based on all the cases with valid data for that pair.
Syntax		CORRELATIONS /VARIABLES=mobility_pc_now_severity ADL_now_level communication_now_level functional3items /PRINT=TWOTAIL NOSIG /MISSING=PAIRWISE.
Resources	Processor Time	00:00:00.000
	Elapsed Time	00:00:00.004

```
[DataSet1] C:\Users\CNMI.DESKTOP-R9ODJFL\Desktop\YRS for MAI.sav
```

Correlations

		mobility_pc_now_severity	ADL_now_level
mobility_pc_now_severity	Pearson Correlation	1	.773**
	Sig. (2-tailed)		.000
	N	337	337
ADL_now_level	Pearson Correlation	.773**	1
	Sig. (2-tailed)	.000	
	N	337	337
communication_now_level	Pearson Correlation	.038	.387**
	Sig. (2-tailed)	.493	.000
	N	337	337
functional3items	Pearson Correlation	.883**	.957**
	Sig. (2-tailed)	.000	.000
	N	337	337

**. Correlation is significant at the 0.01 level (2-tailed).

Correlations

		communication_now_level	functional3items
mobility_pc_now_severity	Pearson Correlation	.038	.883**
	Sig. (2-tailed)	.493	.000
	N	337	337
ADL_now_level	Pearson Correlation	.387**	.957**
	Sig. (2-tailed)	.000	.000
	N	337	337
communication_now_level	Pearson Correlation	1	.427**
	Sig. (2-tailed)		.000
	N	337	337
functional3items	Pearson Correlation	.427**	1
	Sig. (2-tailed)	.000	
	N	337	337

**. Correlation is significant at the 0.01 level (2-tailed).

```
CORRELATIONS
  /VARIABLES=symptom10items functional3items overallhealth_now_level Total14it
ems
  /PRINT=TWOTAIL NOSIG
  /MISSING=PAIRWISE.
```

Correlations

subscale-subscale & subscale-total

Notes

Output Created		28-Nov-2022 16:30:55
Comments		
Input	Data	C:\Users\CNMI.DESKTOP-R9ODJFL\Desktop\YRS for MAI.sav
	Active Dataset	DataSet1
	Filter	<none>
	Weight	<none>
	Split File	<none>
	N of Rows in Working Data File	337
Missing Value Handling	Definition of Missing	User-defined missing values are treated as missing.
	Cases Used	Statistics for each pair of variables are based on all the cases with valid data for that pair.
Syntax		CORRELATIONS /VARIABLES=symptom10items functional3items overallhealth_now_level Total14items /PRINT=TWOTAIL NOSIG /MISSING=PAIRWISE.
Resources	Processor Time	00:00:00.000
	Elapsed Time	00:00:00.005

```
[DataSet1] C:\Users\CNMI.DESKTOP-R9ODJFL\Desktop\YRS for MAI.sav
```

Correlations

		symptom10items	functional3items
symptom10items	Pearson Correlation	1	.594**
	Sig. (2-tailed)		.000
	N	337	337
functional3items	Pearson Correlation	.594**	1
	Sig. (2-tailed)	.000	
	N	337	337
overallhealth_now_level	Pearson Correlation	-.055	-.101
	Sig. (2-tailed)	.312	.065
	N	337	337
Total14items	Pearson Correlation	.906**	.741**
	Sig. (2-tailed)	.000	.000
	N	337	337

**. Correlation is significant at the 0.01 level (2-tailed).

Correlations

		overallhealth_now_level	Total14items
symptom10items	Pearson Correlation	-.055	.906**
	Sig. (2-tailed)	.312	.000
	N	337	337
functional3items	Pearson Correlation	-.101	.741**
	Sig. (2-tailed)	.065	.000
	N	337	337
overallhealth_now_level	Pearson Correlation	1	.267**
	Sig. (2-tailed)		.000
	N	337	337
Total14items	Pearson Correlation	.267**	1
	Sig. (2-tailed)	.000	
	N	337	337

**. Correlation is significant at the 0.01 level (2-tailed).

```
COMPUTE Total15items=(dyspnea_rest_now_severity + dyspnea_stair_now_severity +
 larynx_prob_serverity + voice_impact_severity + nutrition_impact_severity + f
atigue_now_severity + painnow_severity + concentration + short_memory + depres
sion_now_level +
mobility_pc_now_severity + ADL_now_level + communication_now_level + overallhe
alth_now_level).
EXECUTE.
CORRELATIONS
  /VARIABLES=symptom10items functional3items overallhealth_now_level Total15it
ems
  /PRINT=TWOTAIL NOSIG
  /MISSING=PAIRWISE.
```

Correlations

subscale-subscale & subscale-total

Notes

Output Created		28-Nov-2022 16:34:40
Comments		
Input	Data	C:\Users\CNMI.DESKTOP-R9ODJFL\Desktop\YRS for MAI.sav
	Active Dataset	DataSet1
	Filter	<none>
	Weight	<none>
	Split File	<none>
	N of Rows in Working Data File	337
Missing Value Handling	Definition of Missing	User-defined missing values are treated as missing.
	Cases Used	Statistics for each pair of variables are based on all the cases with valid data for that pair.
Syntax		CORRELATIONS /VARIABLES=symptom10items functional3items overallhealth_now_level Total15items /PRINT=TWOTAIL NOSIG /MISSING=PAIRWISE.
Resources	Processor Time	00:00:00.000
	Elapsed Time	00:00:00.006

```
[DataSet1] C:\Users\CNMI.DESKTOP-R9ODJFL\Desktop\YRS for MAI.sav
```

Correlations

		symptom10items	functional3items
symptom10items	Pearson Correlation	1	.594**
	Sig. (2-tailed)		.000
	N	337	337
functional3items	Pearson Correlation	.594**	1
	Sig. (2-tailed)	.000	
	N	337	337
overallhealth_now_level	Pearson Correlation	-.055	-.101
	Sig. (2-tailed)	.312	.065
	N	337	337
Total15items	Pearson Correlation	.906**	.741**
	Sig. (2-tailed)	.000	.000
	N	337	337

**. Correlation is significant at the 0.01 level (2-tailed).

Correlations

		overallhealth_now_level	Total15items
symptom10items	Pearson Correlation	-.055	.906**
	Sig. (2-tailed)	.312	.000
	N	337	337
functional3items	Pearson Correlation	-.101	.741**
	Sig. (2-tailed)	.065	.000
	N	337	337
overallhealth_now_level	Pearson Correlation	1	.267**
	Sig. (2-tailed)		.000
	N	337	337
Total15items	Pearson Correlation	.267**	1
	Sig. (2-tailed)	.000	
	N	337	337

**. Correlation is significant at the 0.01 level (2-tailed).

```
DESCRIPTIVES VARIABLES=overallhealth_now_level symptom10items functional3items
  /STATISTICS=MEAN STDDEV RANGE MIN MAX.
```

Descriptives

Notes

Output Created		28-Nov-2022 16:39:30
Comments		
Input	Data	C:\Users\CNMI.DESKTOP-R9ODJFL\Desktop\YRS for MAI.sav
	Active Dataset	DataSet1
	Filter	<none>
	Weight	<none>
	Split File	<none>
	N of Rows in Working Data File	337
Missing Value Handling	Definition of Missing	User defined missing values are treated as missing.
	Cases Used	All non-missing data are used.
Syntax		DESCRIPTIVES VARIABLES=overallhealth_now_level symptom10items functional3items /STATISTICS=MEAN STDDEV RANGE MIN MAX.
Resources	Processor Time	00:00:00.000
	Elapsed Time	00:00:00.004

```
[DataSet1] C:\Users\CNMI.DESKTOP-R9ODJFL\Desktop\YRS for MAI.sav
```

Descriptive Statistics

	N	Range	Minimum	Maximum	Mean	Std. Deviation
overallhealth_now_level	337	10.0	.0	10.0	8.047	2.5804
symptom10items	337	64.00	.00	64.00	2.0890	5.42133
functional3items	337	20.00	.00	20.00	.5282	2.64236
Valid N (listwise)	337					

```
RELIABILITY
  /VARIABLES=dyspnea_rest_now_severity dyspnea_stair_now_severity larynx_prob_
serverity voice_impact_severity nutrition_impact_severity mobility_pc_now_seve
rity fatigue_now_severity ADL_now_level painnow_severity concentration short_m
emory
communication_now_level depression_now_level overallhealth_now_level
  /SCALE('ALL VARIABLES') ALL
  /MODEL=ALPHA
  /STATISTICS=DESCRIPTIVE SCALE
  /SUMMARY=TOTAL.
```

Reliability Final C19

YRS 14 items

Notes

Output Created		28-Nov-2022 16:42:28
Comments		
Input	Data	C:\Users\CNMI.DESKTOP-R9ODJFL\Desktop\YRS for MAI.sav
	Active Dataset	DataSet1
	Filter	<none>
	Weight	<none>
	Split File	<none>
	N of Rows in Working Data File	337
	Matrix Input	
Missing Value Handling	Definition of Missing	User-defined missing values are treated as missing.
	Cases Used	Statistics are based on all cases with valid data for all variables in the procedure.
Syntax		RELIABILITY<br>/VARIABLES=dyspnea_rest_now_severity dyspnea_stair_now_severity larynx_prob_serverity voice_impact_severity nutrition_impact_severity mobility_pc_now_severity fatigue_now_severity ADL_now_level painnow_severity concentration short_memory communication_now_level depression_now_level overallhealth_now_level<br>/SCALE('ALL VARIABLES') ALL<br>/MODEL=ALPHA<br>/STATISTICS=DESCRIPTIVE SCALE<br>/SUMMARY=TOTAL.
Resources	Processor Time	00:00:00.000
	Elapsed Time	00:00:00.005

```
[DataSet1] C:\Users\CNMI.DESKTOP-R9ODJFL\Desktop\YRS for MAI.sav
```

Scale: ALL VARIABLES

Case Processing Summary

		N	%
Cases	Valid	337	100.0
	Excluded[a]	0	.0
	Total	337	100.0

a. Listwise deletion based on all variables in the procedure.

Reliability Statistics

Cronbach's Alpha	N of Items
.723	14

Item Statistics

	Mean	Std. Deviation	N
dyspnea_rest_now_severity	.092	.5122	337
dyspnea_stair_now_severity	.377	1.2502	337
larynx_prob_serverity	.134	.7500	337
voice_impact_severity	.047	.4406	337
nutrition_impact_severity	.101	.5361	337
mobility_pc_now_severity	.252	1.3224	337
fatigue_now_severity	.718	1.5662	337
ADL_now_level	.226	1.2804	337
painnow_severity	.205	.9143	337
concentration	.095	.6878	337
short_memory	.196	.9307	337
communication_now_level	.050	.5828	337
depression_now_level	.125	.9493	337
overallhealth_now_level	8.047	2.5804	337

Item-Total Statistics

	Scale Mean if Item Deleted	Scale Variance if Item Deleted	Corrected Item-Total Correlation	Cronbach's Alpha if Item Deleted
dyspnea_rest_now_severity	10.573	53.287	.479	.707
dyspnea_stair_now_severity	10.288	52.027	.197	.725
larynx_prob_serverity	10.531	52.000	.423	.704
voice_impact_severity	10.617	53.273	.570	.705
nutrition_impact_severity	10.564	52.645	.540	.703
mobility_pc_now_severity	10.412	47.457	.435	.694
fatigue_now_severity	9.947	43.289	.553	.675
ADL_now_level	10.439	46.164	.536	.681
painnow_severity	10.460	48.457	.616	.682
concentration	10.570	51.359	.538	.697
short_memory	10.469	48.905	.566	.686
communication_now_level	10.614	51.898	.583	.698
depression_now_level	10.540	50.243	.445	.698
overallhealth_now_level	2.617	53.392	-.077	.837

Scale Statistics

Mean	Variance	Std. Deviation	N of Items
10.665	57.134	7.5587	14

```
RELIABILITY
  /VARIABLES=dyspnea_rest_now_severity dyspnea_stair_now_severity larynx_prob_
serverity voice_impact_severity nutrition_impact_severity fatigue_now_severity
 painnow_severity concentration short_memory depression_now_level
  /SCALE('ALL VARIABLES') ALL
  /MODEL=ALPHA
  /STATISTICS=DESCRIPTIVE SCALE
  /SUMMARY=TOTAL.
```

Reliability final

symptom severity subscale

Notes

Output Created		28-Nov-2022 16:43:28
Comments		
Input	Data	C:\Users\CNMI.DESKTOP-R9ODJFL\Desktop\YRS for MAI.sav
	Active Dataset	DataSet1
	Filter	<none>
	Weight	<none>
	Split File	<none>
	N of Rows in Working Data File	337
	Matrix Input	
Missing Value Handling	Definition of Missing	User-defined missing values are treated as missing.
	Cases Used	Statistics are based on all cases with valid data for all variables in the procedure.
Syntax		RELIABILITY /VARIABLES=dyspnea_rest_now_severity dyspnea_stair_now_severity larynx_prob_serverity voice_impact_severity nutrition_impact_severity fatigue_now_severity painnow_severity concentration short_memory depression_now_level /SCALE('ALL VARIABLES') ALL /MODEL=ALPHA /STATISTICS=DESCRIPTIVE SCALE /SUMMARY=TOTAL.
Resources	Processor Time	00:00:00.000
	Elapsed Time	00:00:00.005

```
[DataSet1] C:\Users\CNMI.DESKTOP-R9ODJFL\Desktop\YRS for MAI.sav
```

Scale: ALL VARIABLES

Case Processing Summary

		N	%
Cases	Valid	337	100.0
	Excluded[a]	0	.0
	Total	337	100.0

a. Listwise deletion based on all variables in the procedure.

Reliability Statistics

Cronbach's Alpha	N of Items
.794	10

Item Statistics

	Mean	Std. Deviation	N
dyspnea_rest_now_severity	.092	.5122	337
dyspnea_stair_now_severity	.377	1.2502	337
larynx_prob_serverity	.134	.7500	337
voice_impact_severity	.047	.4406	337
nutrition_impact_severity	.101	.5361	337
fatigue_now_severity	.718	1.5662	337
painnow_severity	.205	.9143	337
concentration	.095	.6878	337
short_memory	.196	.9307	337
depression_now_level	.125	.9493	337

Item-Total Statistics

	Scale Mean if Item Deleted	Scale Variance if Item Deleted	Corrected Item-Total Correlation	Cronbach's Alpha if Item Deleted
dyspnea_rest_now_severity	1.997	26.307	.537	.777
dyspnea_stair_now_severity	1.712	24.938	.231	.817
larynx_prob_serverity	1.955	25.120	.493	.774
voice_impact_severity	2.042	26.522	.589	.777
nutrition_impact_severity	1.988	26.518	.468	.781
fatigue_now_severity	1.371	19.300	.555	.778
painnow_severity	1.884	22.793	.660	.752
concentration	1.994	24.934	.580	.767
short_memory	1.893	23.370	.573	.762
depression_now_level	1.964	23.725	.515	.769

Scale Statistics

Mean	Variance	Std. Deviation	N of Items
2.089	29.391	5.4213	10

```
RELIABILITY
  /VARIABLES=mobility_pc_now_severity ADL_now_level communication_now_level
  /SCALE('ALL VARIABLES') ALL
```

```
/MODEL=ALPHA
/STATISTICS=DESCRIPTIVE SCALE
/SUMMARY=TOTAL.
```

Reliability final

functional ability subscale

Notes

Output Created		28-Nov-2022 16:44:58
Comments		
Input	Data	C:\Users\CNMI.DESKTOP-R9ODJFL\Desktop\YRS for MAI.sav
	Active Dataset	DataSet1
	Filter	<none>
	Weight	<none>
	Split File	<none>
	N of Rows in Working Data File	337
	Matrix Input	
Missing Value Handling	Definition of Missing	User-defined missing values are treated as missing.
	Cases Used	Statistics are based on all cases with valid data for all variables in the procedure.
Syntax		RELIABILITY /VARIABLES=mobility_pc_now_severity ADL_now_level communication_now_level /SCALE('ALL VARIABLES') ALL /MODEL=ALPHA /STATISTICS=DESCRIPTIVE SCALE /SUMMARY=TOTAL.
Resources	Processor Time	00:00:00.000
	Elapsed Time	00:00:00.005

```
[DataSet1] C:\Users\CNMI.DESKTOP-R9ODJFL\Desktop\YRS for MAI.sav
```

Scale: ALL VARIABLES

Case Processing Summary

		N	%
Cases	Valid	337	100.0
	Excluded[a]	0	.0
	Total	337	100.0

a. Listwise deletion based on all variables in the procedure.

Reliability Statistics

Cronbach's Alpha	N of Items
.699	3

Item Statistics

	Mean	Std. Deviation	N
mobility_pc_now_severity	.252	1.3224	337
ADL_now_level	.226	1.2804	337
communication_now_level	.050	.5828	337

Item-Total Statistics

	Scale Mean if Item Deleted	Scale Variance if Item Deleted	Corrected Item-Total Correlation	Cronbach's Alpha if Item Deleted
mobility_pc_now_severity	.276	2.558	.633	.452
ADL_now_level	.303	2.146	.852	.054
communication_now_level	.478	6.006	.223	.872

Scale Statistics

Mean	Variance	Std. Deviation	N of Items
.528	6.982	2.6424	3
